# Sites of gastrointestinal lesion induced by mycophenolate mofetil: a comparison with enteric-coated mycophenolate sodium in rats

**DOI:** 10.1186/s40360-018-0234-1

**Published:** 2018-07-04

**Authors:** Yichen Jia, Rulin Wang, Long Li, Ying Zhang, Jiawei Li, Jina Wang, Xuanchuan Wang, Guisheng Qi, Ruiming Rong, Ming Xu, Tongyu Zhu

**Affiliations:** 1 Department of Urology, Shanghai Key laboratory of Organ Transplantation, Zhongshan Hospital, Fudan University, 180 Fenglin Road, Shanghai, 200032 People’s Republic of China; 2 Department of Urology, The First Affiliated Hospital of Zhengzhou University, Zhengzhou, 450052 People’s Republic of China; 3 Department of Pathology, Xin Hua Hospital Affiliated to Shanghai Jiao Tong University School of Medicine, Shanghai, 200092 People’s Republic of China

**Keywords:** Enteric-coated mycophenolate sodium, Mycophenolate mofetil, GI side effects, Pharmacokinetics

## Abstract

**Background:**

Immunosuppressant drugs for renal transplant mycophenolate mofetil (MMF) and enteric-coated mycophenolate sodium (EC-MPS) cause gastrointestinal (GI) disorders. The specific site of GI tract targeted by MMF and EC-MPS remains unclear.

**Methods:**

In this study, we investigated the effects of MMF and EC-MPS on stomach, duodenum, jejunum, ileum, colon and rectum using a rat model. Rats were randomized into five groups: control, MMF (100 mg/kg·d), mofetil (30 mg/kg·d), EC-MPS (72 mg/Kg·d), mofetil + EC-MPS. Each group was treated with drugs once a day for 7 days through intra-gastric gavage. Diarrhea grade of each rat were measured every day, as well as the body weight. Blood was collected by tail nick and Seven days later, the rats were sacrificed, GI tissues were collected for Histological research.

**Results:**

The results showed that diarrhea grade and weight loss were significantly higher in MMF group than other groups. The pathological score of MMF group was significantly higher than EC-MPS group and EC-MPS + mofetil group in jejunum and ileum tissues, but not other segments of GI tract. Absorption of EC-MPS is delayed, compared to that of MMF. MPAG concentration in duodenum, jejunum and ileum tissues of MMF group is higher than EC-MPS group. Mofetil may increase the magnitude of MPA absorption.

**Conclusions:**

Our data suggested that MMF might target jejunum and ileum and induce GI injury. EC-MPS causes less injury in GI tract than MMF, probably due to its kinetic property.

## Background

Mycophenolate mofetil (MMF) and enteric-coated mycophenolate sodium (EC-MPS) are currently and commonly used mycophenolate compounds as adjunct immunosuppressants in renal transplantation [1, 2]. MMF is made from mycophenolic acid (MPA) by ester with N-(2-hydroxyethyl) morpholine (mofetil). It is an immediate-release formulation of MPA and absorbed in the stomach and small intestine. It is hydrolyzed to produce MPA and mofetil after absorption in the stomach and the proximal small intestine [1, 3]. EC-MPS does not have mofetil. EC-MPS is a delayed-release formulation of mycophenolate sodium with enteric-coating. The enteric coating is dissolved at pH 5.5 to 6.0, allowing for delayed MPA delivery until it reaches the small intestine [4, 5].

Mycophenolic acid (MPA) is a nonnucleoside, noncompetitive, potent, selective, and reversible inhibitor of inosine monophosphate dehydrogenase (IMPDH), which is the rate-limiting enzyme in the de novo synthesis pathway of guanosine triphosphate (GTP) [6]. Both T and B lymphocytes are highly dependent on the generation of GTP, therefore, MPA may arrest T- and B-lymphocyte proliferation and is effectively and routinely used as an adjunct immunosuppressant in renal transplantation [7–9]. MPA is primarily metabolized to a phenolic glucuronide MPAG in rats and humans through glucuronidation [10]. MPAG is excreted by the transporter MRP2 (ABCC2) into the bile and subsequently cleaved by β-glucuronidase, regenerating MPA which is reabsorbed in intestine. This enterohepatic circulation recycles MPA, prolonging the half-life of MPA and increasing intestinal exposure of MPA.

MPA therapy is associated with GI adverse events [11–13]. The incidence of MPA-related GI adverse events ranges from 45 to 80% in recipients [14–16]. In animal models, MPA may cause mucosal ulceration, erosion, and necrosis of stomach and intestine. Clinically, MPA-related GI toxicity affects the GI tract at various points, with evidence of villous atrophy of the duodenum and erosive enterocolitis of both the small and large intestines with a presentation similar to Crohn’s disease [17, 18]. One study of more than 400 de novo renal transplant patients indicated that lower GI complications are slightly less common than upper GI events [19].

MMF cause significant GI complications, including nausea, vomiting, ulcers, gastritis, diarrhea, and abdominal pain [11, 20]. Clinical studies showed that MMF caused gastritis, diarrhea, and anorexia in a dose-dependent manner. Because of this, the dosing of MMF is reduced and interrupted, even discontinued, increasing the risk of acute rejection or graft loss [21–23]. EC-MPS is designed to reduce MPA-caused GI complication [24]. Although the mechanism underlying MPA-induced GI side effects is not completely clear, a clinical study shows that EC-MPS has less effects on GI tract than MMF does with combined immunosuppressive regimens [25]. Although both MMF and EC-MPS may cause diarrhea, nausea, vomiting, gastroesophageal reflux disease, and abdominal pain [26, 27], the susceptible sites of the GI tract to MMF and EC-MPS remains unclear.

In this study, we investigated the toxic effects of MMF and EC-MPS on six anatomical segments of GI tract using a rat model, including stomach, duodenum, jejunum, ileum, colon, and rectum. We confirmed that MMF-treated rats are more susceptible to MPA GI toxicity than EC-MPS-treated rats. MMF might target jejunum and ileum and induce GI injury. EC-MPS causes less injury in GI tract that MMF, probably due to it kinetic property.

## Methods

### Experimental animals

Male Sprague Dawley rats were purchased from The SLAC Laboratory Animal Center (Shanghai, China). The rats were maintained under specific pathogen-free conditions, which were housed in a local facility for laboratory animal care and fed a standard diet and water. Rats weighing 180 to 230 g were used for the experiments. Animals were held in plastic cages with hardwood chips. They were provided with food and water ad libitum. The experimental protocol was approved by the Committee of Animal Care of Fudan University.

### Dosing regimens and sample collection

MMF was purchased from Roche Pharmaceuticals Co. (Shanghai, China), and made into powder. Mofetil was purchased from Sinopharm Chemical Reagent Co. (Shanghai, China). EC-MPS (Novartis Pharma Stein AG) was made into enteric-coat microcapsule of mycophenolate sodium by School of Pharmacy Fudan University. Fourty rats were randomly assigned into five groups (each group, *n* = 8): (1) the control group, which was given normal saline (NS); (2) the MMF group, which was given 100 mg/kg·d MMF; (3) the EC-MPS group, which was given 72 mg/kg·d EC-MPS; (4) the mofetil group, which was given 30 mg/kg·d mofetil; and (5) EC-MPS + mofetil group, which was given 72 mg/kg·d EC-MPS and 30 mg/kg·d mofetil. This is designed to evaluate the contribution of mofetil to the effect of MMF and EC-MPS on MPA GI complication. The drugs were diluted in NS and used to treat rats through oral gavage for 14 consecutive days. 100 mg MMF, 72 mg EC-MPS were equivalent to 50 mg MPA.

Following the last treatment, blood (100 μl) was collected at 0, 15, 30, 60, 120, and 240 min by tail nick for determination of MPA and MPAG pharmacokinetic profiles. The rats were sacrificed with an intraperitoneal injection of urethane (1.5 g/kg), GI tissues including stomach, duodenum, jejunum, ileum, colon, and rectum were collected and each was divided into two parts. One part was homogenized and the other part was fixed in 10% formalin for histological analysis. In each animal (*n* = 8 for each group), one sample of every GI site was collected for histologic analysis.

Gastrointestinal segments were defined as follows: stomach, the part close to pyloric sphincter; duodenum, at the site 1 cm below pyloric sphincter to ligament of Trietz; upper jejunum, at the site 5 cm below stomach (upper half of remaining small intestine); lower jejunum-ileum, at the site 5 cm above cecum (lower half of small intestine); colon, at the site 5 cm below ileum-cecum (cecum to rectum); and rectum, at the site 3 cm above anus [28, 29].

### Determination of MPA and MPAG in plasma and GI tissue

Plasma and tissue MPA and MPAG were determined using a high-performance liquid chromatography (HPLC) with ultraviolet detection [30]. Briefly, plasma and tissue homogenates were precipitated with acetonitrile, spiked with propafenone hydrochloride as an internal standard (50 μg/ml in sample). MPA and MPAG were determined using liquid chromatography/mass spectrometry (LC/MS). The HPLC conditions included a C18 column (150*4.6 mm; Kromasil, AkzoNobel, Sweden), isocratic mobile phase [46% methanol: 54% aqueous trifluoroacetic acid (0.1%; pH 2.5)]. Analysis was performed under a 20 μl injection, solvent flow of 1.5 ml/min, total run time of 15 min per injection, and UV detection at 295 nm. The appropriate standard curves for MPA and MPAG were linear over the range of 0.5-100 μg/ml and 2.5-100 μg/ml, respectively.

Non-compartmental model (linear trapezoidal model) was used to calculate pre-dose concentration (C_0_), maximum concentration (C_max_), and area under the plasma concentration-time curve from 0 to 240 min.

### Assessment of diarrhea grade and body weight

Body weight and stools were monitored daily. Food intake was not assessed. Stools were graded for degree of diarrhea by the following scale (0, firm stool; 1, malformed stool; 2, watery stool with perianal staining; 3, severe perianal staining).

### Histological analysis

Tissue samples fixed in 10% formalin were processed for histological examination following the standard procedure. The tissues in formalin were embedded in paraffin and cut 5-6 μm sections using rotary microtome. The sections were stained with hematoxylin and eosin and analyzed under light microscope [31].

The histological changes in the GI tract was scored using a semi-quantitative scale. The gastric injury were graded from 0 to 5 (0, no lesions; 1, lesions seen in mucosal surface, no damaged gastric pit cell; 2, damages with gastric pits, no damaged gastric gland cell; 3, lesions of gastric gland cells; 4, partial mucosal necrosis, multiple linear ulcer and hemorrhage; 5, total mucosal necrosis). The intestinal injury were graded from 0 to 5 according to Criteria of Chiu grading (0, normal mucosa villi; 1, development of subepithelial Gruenhagen’s space, usually at the apex of the villus and often with capillary congestion; 2, extension of the space with moderate lifting of epithelial layer from the lamina propria; 3, massive epithelial lifting with a few denuded villi; 4, denuded villi with exposed capillaries; 5, disintegration of the lamina propria, ulceration and hemorrhage) [32]. The histopathological studies were conducted by a pathologist who was blinded to the study.

### Statistical analysis

The data are presented as the mean ± standard deviation (SD). Statistical differences between groups were analyzed using one way analysis of variance test. Histological data were analyzed using χ^2^ test. Differences were considered statistically significant if the *p* value was less than 0.05.

## Results

### The pharmacokinetics of EC-MPS and MMF

We analyzed the pharmacokinetics of EC-MPS and MMF by determining the plasma MPA and MPAG profiles. The results were listed in Table 1. There was no significant difference in the AUC values for MPA or MPAG among EC-MPS, MMF and EC-MPS + mofetil groups. The maximum plasma MPA concentrations (C_max_) were similar between MMF and EC-MPS + mofetil groups, and they were significantly higher than that of the EC-MPS group. The time to reach maximal plasma MPA concentration (T_max_) were similar between EC-MPS and EC-MPS + mofetil groups, and they were significantly longer than that of the MMF group. The results suggested that clearance of EC-MPS and MMF was the same among these three groups. Absorption of EC-MPS instead of MMF is delayed. Mofetil may increase the magnitude of MPA absorption.

**Table 1:** Serum MPA and MPAG pharmacokinetics

Pharmacokinetic parameters	MPA			MPAG		
	EC-MPS	MMF	EC-MPS + mofetil	EC-MPS	MMF	EC-MPS + mofetil
AUC (μg•min/ml)	69.30	88.25	73.49	91.53	71.94	88.67
C_max_ (μg/ml)	21.74	40.74	40.45	30.56	19.12	43.98
t_max_ (min)	60	30	60	120	120	120

The maximum plasma MPAG concentrations (C_max_) were similar between EC-MPS and EC-MPS + mofetil groups, and they were significantly higher than that of the MMF group. The time to maximal concentration (T_max_) of MPAG were similar among EC-MPS, MMF, and EC-MPS + mofetil groups. The results suggested that the glucuronidation of EC-MPS was higher in magnitude and delayed than that of MMF. Mofetil may not affect the glucuronidation.

### EC-MPS decrease diarrhea grade and increase body weight

**Fig. 1:**
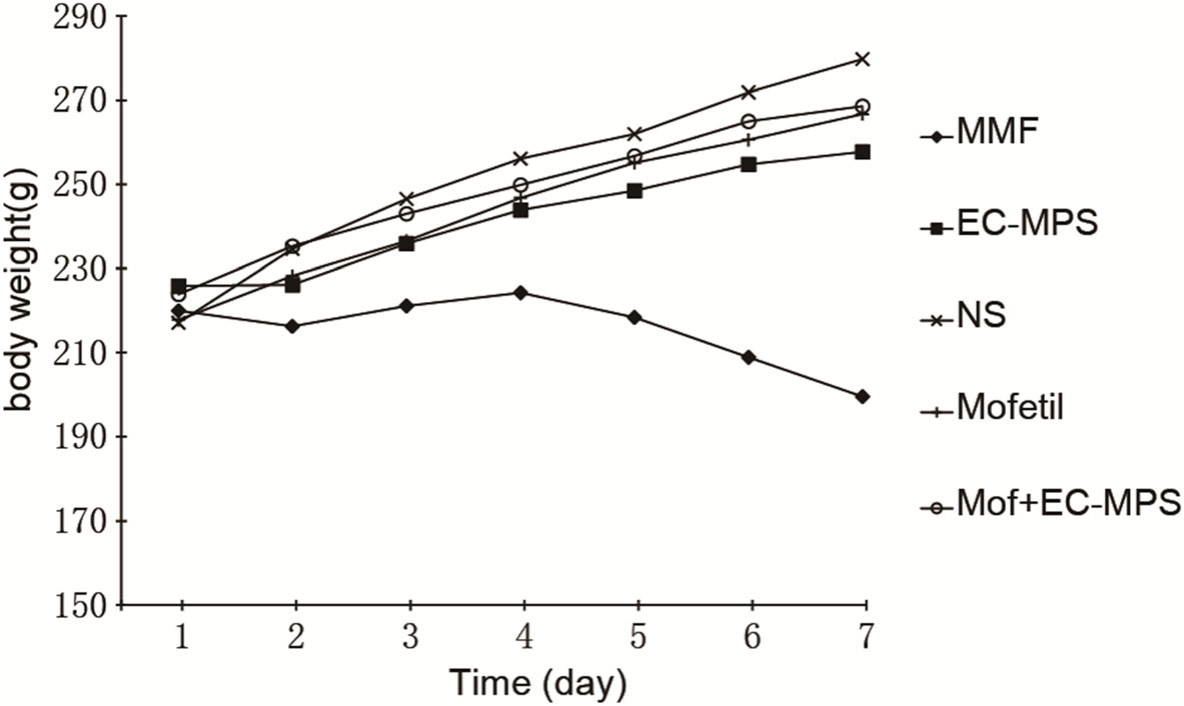
The effects of MMF and EC-MPS on the body weight. Treatment of rats with MMF resulted in significant more loss of body weight than control group (199.5 ± 8.3 g vs 279.8 ± 7.5, *p <* 0.05) and EC-MPS treatment group (199.5 ± 8.3 g vs 257.8 ± 9.6, *p <* 0.05)

**Fig. 2:**
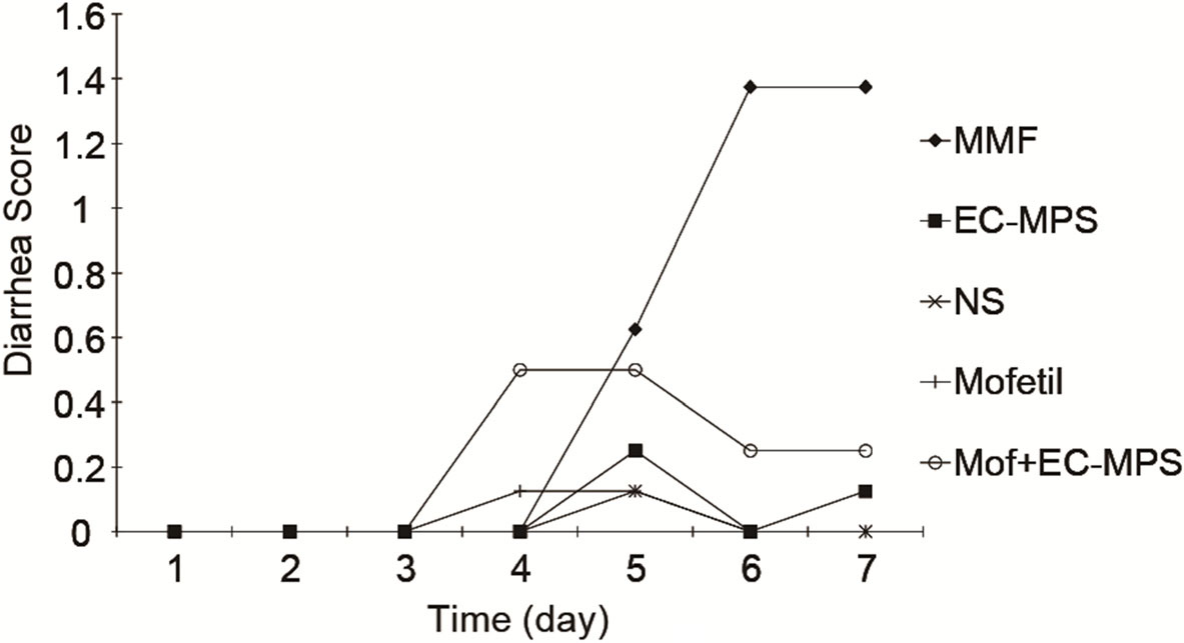
The effects of MMF and EC-MPS on the diarrhea score. Treatment of rats with MMF resulted in significant increases in the diarrhea score, compared with the control group (1.375 ± 0.34 vs 0 ± 0*, p <* 0.05) and EC-MPS treatment group (1.375 ± 0.34 vs 0.125 ± 0.04*, p <* 0.05)

Treatment of rats with MMF resulted in significant more loss of body weight than control group (199.5 ± 8.3 g vs 279.8 ± 7.5*, p <* 0.05) and EC-MPS treatment group (199.5 ± 8.3 g vs 257.8 ± 9.6*, p <* 0.05) (Fig. 1). Treatment of rats with MMF resulted in significant increases in the diarrhea score, compared with the control group (1.375 ± 0.34 vs 0 ± 0*, p <* 0.05) and EC-MPS treatment group (1.375 ± 0.34 vs 0.125 ± 0.04*, p <* 0.05) (Fig. 2). There was no obvious differences in the diarrhea score between the control group and EC-MPS treatment group (Fig. 2).

### EC-MPS protect jejunum and ileum segments in histological analysis

**Fig. 3:**
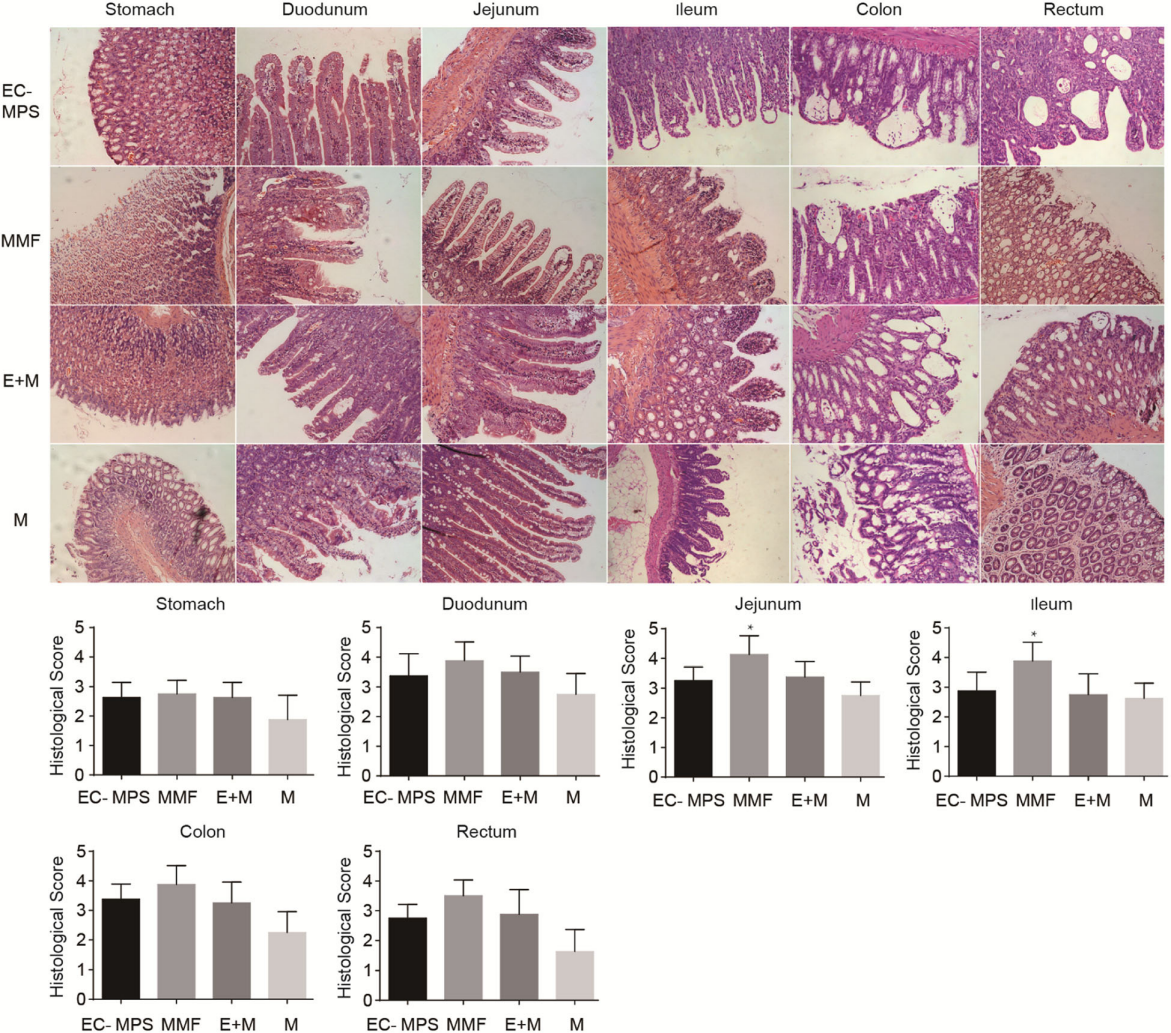
Comparisons of the histological scores in GI tract. Comparisons of the histological scores in stomach, duodenum, jejunum, ileum, colon, and rectum among rats treated with indicated drugs in (1) EC-MPS group; (2) MMF group; (3) EC-MPS + mofetil (E + M) group; (4) mofetil (M) group. The histopathological score of the jejunum and ileum segments were significantly higher in the MMF group than the EC-MPS group (4.1 ± 0.3 vs 3.2 ± 0.15*, p <* 0.05) and EC-MPS + mofetil group (4.1 ± 0.3 vs 3.3 ± 0.18*, p <* 0.05). There was no significant difference in the histopathological score of the other tissues among MMF group, EC-MPS group, and EC-MPS + mofetil group

We performed a histopathological analysis on histological changes in GI tissues of each group. The results showed that the histopathological score of the jejunum and ileum segments were significantly higher in the MMF group than the EC-MPS group (4.1 ± 0.3 vs 3.2 ± 0.15*, p <* 0.05) and EC-MPS + mofetil group (4.1 ± 0.3 vs 3.3 ± 0.18*, p <* 0.05) (Fig. 3). There was no significant difference in the histopathological score of the other tissues among MMF group, EC-MPS group, and EC-MPS + mofetil group (Fig. 3).

### EC-MPS decrease the MPAG contents in the GI tissues

**Fig. 4:**
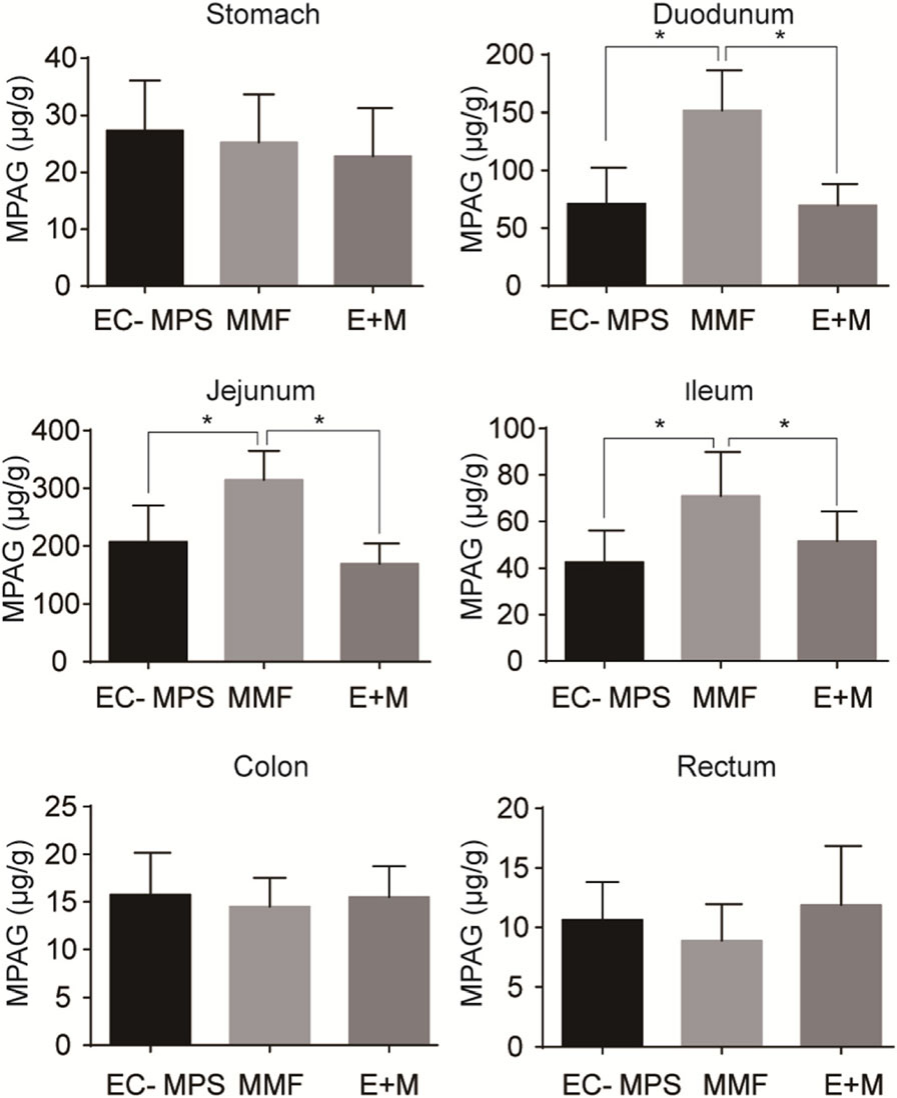
Comparisons of the MPAG levels in GI tract. Comparisons of the MPAG levels in stomach, duodenum, jejunum, ileum, colon, and rectum among rats treated with indicated drugs in (1) EC-MPS group; (2) MMF group; (3) EC-MPS + mofetil (E + M) group. MPAG levels in duodenum, jejunum, and ileum tissues were significantly higher in the MMF group than EC-MPS group (152.4 ± 24.34 vs 72.3 ± 24.23*, p <* 0.05; 312.3 ± 40.34 vs 208.5 ± 47.34*, p <* 0.05; 71.2 ± 28.22 vs 42.2 ± 16.43*, p <* 0.05) and EC-MPS+ mofetil group (152.4 ± 24.34 vs 72.1 ± 15.23*, p <* 0.05; 312.3 ± 40.34 vs 180.7 ± 21.14*, p <* 0.05; 71.2 ± 28.22 vs 52.4 ± 17.15*, p <* 0.05). There was no significant difference in the MPAG levels in stomach, colon, and rectum tissues among all these three groups. In all GI tissues, there are no significant difference between EC-MPS group and EC-MPS+ mofetil group

We determined the MPAG contents in GI tissues of each group. The results showed that MPAG levels in duodenum, jejunum, and ileum tissues were significantly higher in the MMF group than EC-MPS group (152.4 ± 24.34 vs 72.3 ± 24.23*, p <* 0.05; 312.3 ± 40.34 vs 208.5 ± 47.34*, p <* 0.05;71.2 ± 28.22 vs 42.2 ± 16.43*, p <* 0.05) and EC-MPS+ mofetil group (152.4 ± 24.34 vs 72.1 ± 15.23*, p <* 0.05; 312.3 ± 40.34 vs 180.7 ± 21.14*, p <* 0.05;71.2 ± 28.22 vs 52.4 ± 17.15*, p <* 0.05) (Fig. 4). There was no significant difference in the MPAG levels in stomach, colon, and rectum tissues among all these three groups. In all GI tissues, there are no significant difference between EC-MPS group and EC-MPS+ mofetil group (Fig. 4).

## Discussion

In the current study, we find that treatment of rats with EC-MPS resulted in loss of less body weight and lower diarrhea score than MMF groups, suggesting that EC-MPS causes milder and less GI side effects than MMF. We evaluated the toxic effects of MMF and EC-MPS on stomach, duodenum, jejunum, ileum, colon, and rectum using a rat model. The pathological score of MMF group was significantly higher than EC-MPS group and EC-MPS + mofetil group in jejunum and ileum tissues, but not other segments of GI tract. Therefore, MMF may target jejunum and ileum and induce GI injury.

Our pharmacokinetic data is consistent with our finding that MMF may target jejunum and ileum and induce GI injury and that EC-MPS induced less GI complication in the rat model. We found that absorption of EC-MPS is delayed, compared to that of MMF. This is consistent with the previous study on human population [33, 34]. It is very likely that delayed absorption of MPA may occur more distally in the GI tract with EC-MPS than MMF. Further, we found that MPAG concentration in duodenum, jejunum and ileum tissues of MMF group is higher than EC-MPS group, suggesting that MMF is absorbed in the upper GI tract and the absorption of EC-MPS is increased in the distal GI tract. Increased absorption of MMF is the upper GI tract may result in lesions in jejunum and ileum. It is likely that EC-MPS has less GI complication that MMF because of its pharmacokinetic property.

MMF is a mofetil ester of MPA. The stated goal of adding the mofetil ester to MPA was to improve MPA absorption. However, the addition of this ester was not necessary given that MPA is very well absorbed. The hydrolysis of MMF by in vivo esterases results in release of MPA free acid and mofetil. There is limited evidence that mofetil has local irritative effects. Animal studies on alterations in gut gene expression in the presence of mofetil may provide further insight into the pathobiology of this molecule. Our data indicated that mofetil may increase the magnitude of MPA absorption but not affect the glucuronidation. Furthermore, we found that diarrhea score of EC-MPS + mofetil group is higher than EC-MPS group. Therefore, mofetil may cause GI side effects through enhancing absorption of MPA.

This study had some limitations. In our study, daily lavage was applied to deliver drugs in rats to study the pharmacokinetics of MMF and EC-MPS. However, this animal model has its limitations. Improper gastric lavage may cause mechanical injury of upper digestive tract in rats, which may influence the food intake of rats, and consequently influence the measurement of body weight. Furthermore, the differences of drug metabolism between animals and human may account for the differences in the study of AUC between human and animals. The doses of MMF and EC-MPS need to be verified in future study. Lastly, we have detected the content of MPAG in GI tissues, however detecting MPA as the active compound in GI tissue may provide more information about the pharmacokinetics of MMF and EC-MPS.

## Conclusion

In conclusion,EC-MPS showed more advantages in diarrhea grading and weight loss study. GI pathological examination show that MMF may target jejunum and ileum and induce GI injury. EC-MPS causes less injury in GI tract that MMF, probably due to its kinetic property.

## Abbreviations

AUC: Area under the concentration versus time curve
GI: Gastrointestinal
HPLC: High-performance liquid chromatography
MMF: Mycophenolate mofetil
MPA: Mycophenolic acid
MPAG: Mycophenolic acid phenolic glucuronide
